# Rheumatic Heart Disease with Multiple Systemic Emboli: A Rare Occurrence in a Single Subject

**DOI:** 10.7759/cureus.2964

**Published:** 2018-07-11

**Authors:** Ravi R Pradhan, Ashish Jha, Gaurav Nepal, Manju Sharma

**Affiliations:** 1 Internal Medicine, Institute of Medicine, Tribhuvan University Teaching Hospital, Kathmandu, NPL; 2 Medicine, Institute of Medicine, Tribhuvan University Teaching Hospital, Kathmandu, NPL; 3 Maharajgunj Medical Campus, Tribhuvan University Institute of Medicine, Kathmandu, NPL; 4 Cardiology, Manmohan Cardiothoracic Vascular and Transplant Center, Kathmandu, NPL

**Keywords:** rheumatic heart disease, mitral stenosis, acute ischemic stroke, acute myocardial infarction, acute limb ischemia

## Abstract

Valvular heart disease is one of the more common diseases in low- and middle-income countries, when associated with atrial fibrillation (AF), carries a risk of multisystemic embolizations. We report a case of 37-year-old man with multiple systemic emboli consisting of acute ischemic stroke, acute myocardial infarction, and acute limb ischemia. This is a rare occurrence in a single subject. The patient had a background of rheumatic heart disease (RHD) involving severe mitral stenosis (MS) with AF, who was not compliant with his medications. A computed tomography (CT) scan of the head showed right-sided ischemic stroke involving more than one-third of the middle cerebral artery territory. An electrocardiogram (ECG) showed AF and ST-segment elevation in V4 to V6. Cardiac enzymes were elevated. A transthoracic echocardiogram demonstrated hypokinetic left ventricular anterolateral wall, severe MS, and a left atrial clot. An arterial Doppler of the right lower limb showed an occluding thrombus of the right common femoral artery and right popliteal artery with no flow in color Doppler. Patient adherence to medications in cases of RHD prevents devastating outcomes.

## Introduction

Acute rheumatic fever (ARF) results from the body’s autoimmune response to a throat infection caused by *Streptococcus pyogenes*, a group A beta-hemolytic Streptococcus. Rheumatic heart disease (RHD) refers to the long-term cardiac damage caused by either a single severe episode or multiple recurrent episodes of ARF. RHD involving mitral valve causes inflammation and fibrosis with disruption of the atrial architecture. There is increased left atrial pressure that contributes to left atrial dilatation and increased wall stress predisposing to the development of atrial fibrillation leading to left atrial clot and subsequent systemic embolism.

RHD ranks among the important non-communicable diseases in low- and middle­-income countries, and nearly eradicated in high-income countries. It is a sentinel of social inequality and a physical manifestation of poverty, and thus continues to be a substantial health care challenge in less privileged regions of the world [1]. Nevertheless, it is a cause of significant morbidity and mortality.

This case demonstrates a rare and atypical presentation of RHD with severe mitral stenosis (MS) and atrial fibrillation (AF) leading to multiple systemic emboli, that presented with acute ischemic stroke, acute myocardial infarction, and acute limb ischemia. Early recognition and aggressive management led to a satisfactory outcome in this case.

## Case presentation

A 37-year-old man from the Himalayan region of Nepal presented with swelling of the right leg for ten days and sudden onset weakness of the left half of the body for two days. The swelling of the right leg had an insidious onset and was gradually progressive. The pain disabled the patient to move his right leg. Meanwhile, the patient developed weakness of the left half of the body, predominantly in the upper left limb. The patient also developed slurring of speech and deviation of the face towards the right side. He denied any history of chest pain, diaphoresis, shortness of breath, loin pain, nausea or vomiting. He had decreased urine output and red colored urine and was a non-smoker and non-alcoholic. There was no history of hypertension, chronic kidney disease, and diabetes mellitus in this patient.

On detail inquiry, the patient gave a history of recurrent throat infection during childhood, however, he was not medically managed then. Three years back, when he visited a cardiac centre with complaints of shortness of breath and palpitations, a diagnosis of RHD with severe MS and AF was made. On reviewing the past record of the patient, a significantly elevated serum antistreptolysin O (ASO) titer was seen. He was planned for per-cutaneous trans-mitral commissurotomy (PTMC) by his physician. However, he lost the follow-up and was non-compliant to his medications.

**Figure 1:**
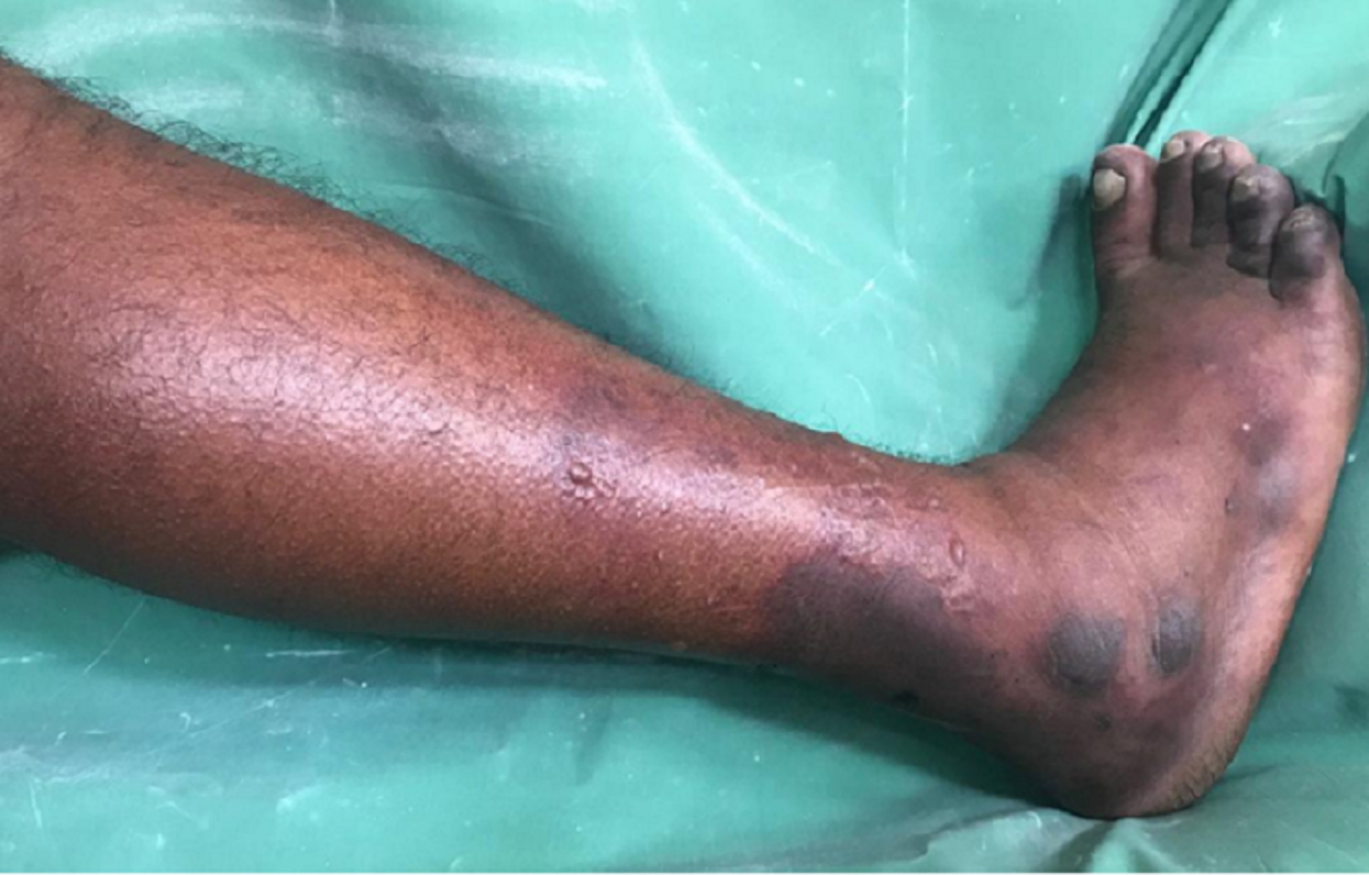
Right leg with swelling below the level of knee and blackish discoloration of toes and lower third of the leg Vesicles could be seen over the lateral aspects of the leg and dorsum of the foot. The limb was cold to touch and tender on palpation. Dorsalis pedis and posterior tibial pulses were not palpable.

On physical examination, his blood pressure measured 110/70 mm Hg; pulse rate was irregularly irregular at 77 beats per minute; respiratory rate was 16 breaths per minute, and body temperature measured 37.1 ^o^C orally. The patient was alert, conscious, and cooperative. There were no appreciable pallor, icterus, clubbing, splinter hemorrhages, rashes and cyanosis. On cardiovascular systemic examination, the first heart sound (S1) was variable in intensity and second heart sound (S2) was normal. There was a low pitched rough rumbling mid-diastolic murmur at the apex heard best when the patient was lying on the left side with breath held in expiration using the bell of the stethoscope. There was a high pitched blowing pansystolic murmur at the left lower sternal border. Examination of the abdomen was unremarkable. On neurological examination, the patient had a left-sided central facial nerve palsy, muscle strengths in the left upper and lower limbs were 2/5 and 3/5 respectively, and there was an ipsilateral Babinski sign. A thorough eye examination along with fundoscopy was unremarkable. Local examination of the right leg revealed swelling below the level of the knee and blackish discoloration of toes and lower third of the leg on inspection. Vesicles could be seen over the lateral aspects of the leg and dorsum of the foot. The limb was cold to touch and tender on palpation. Dorsalis pedis and posterior tibial pulses were not palpable (Figure 1).

**Figure 2:**
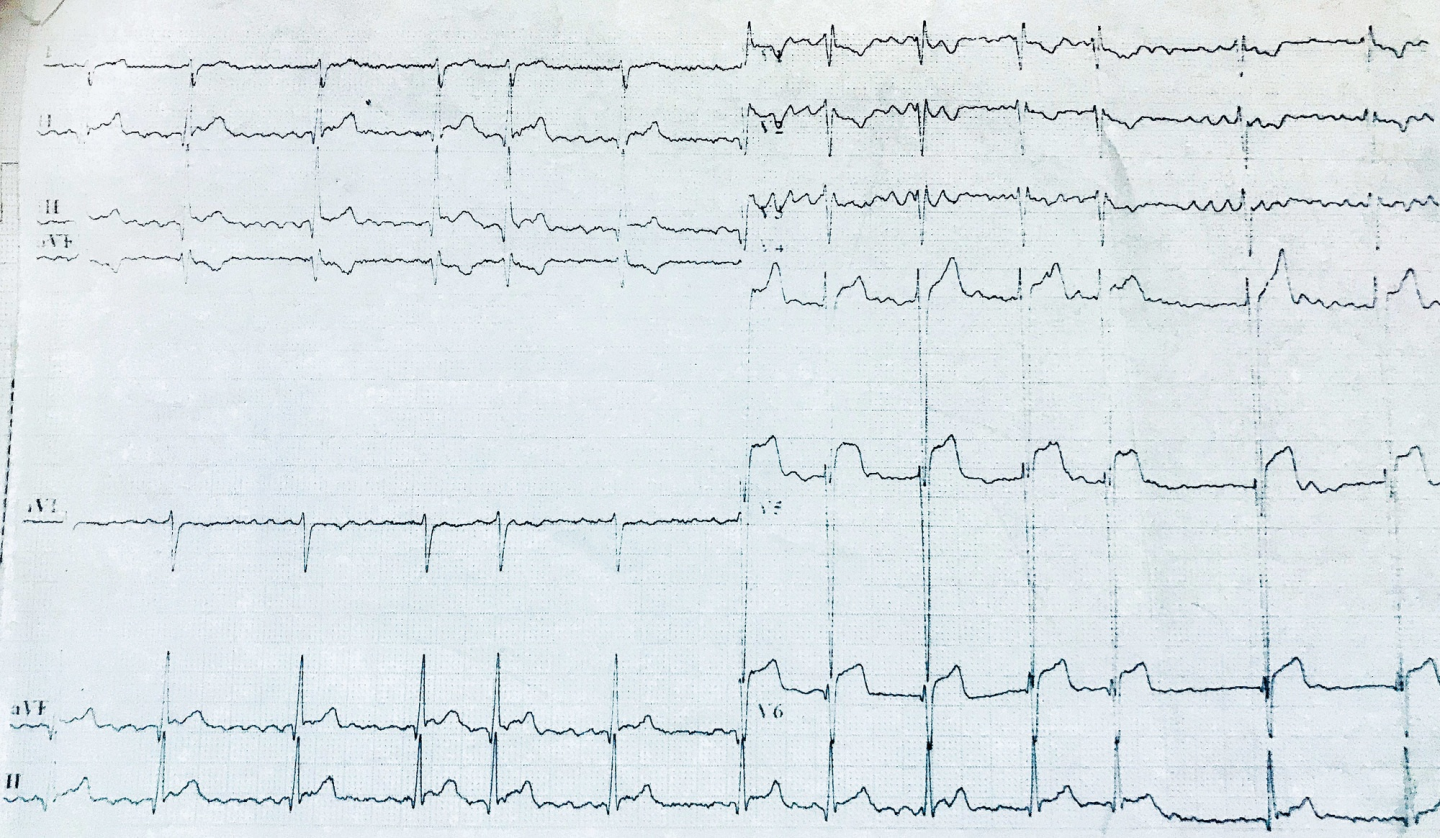
Electrocardiogram showing atrial fibrillation with normal ventricular rate and ST-elevation from V4 to V6

**Figure 3:**
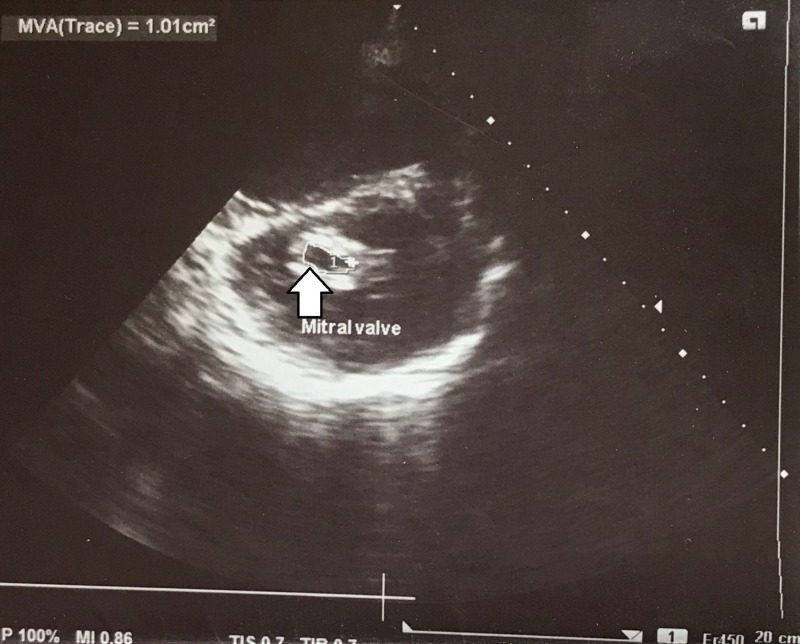
Transthoracic echocardiography on short axis view showing thickened, calcified mitral valve leaflets with MVA of 1.01 cm2 on planometry MVA: mitral valve area

**Figure 4:**
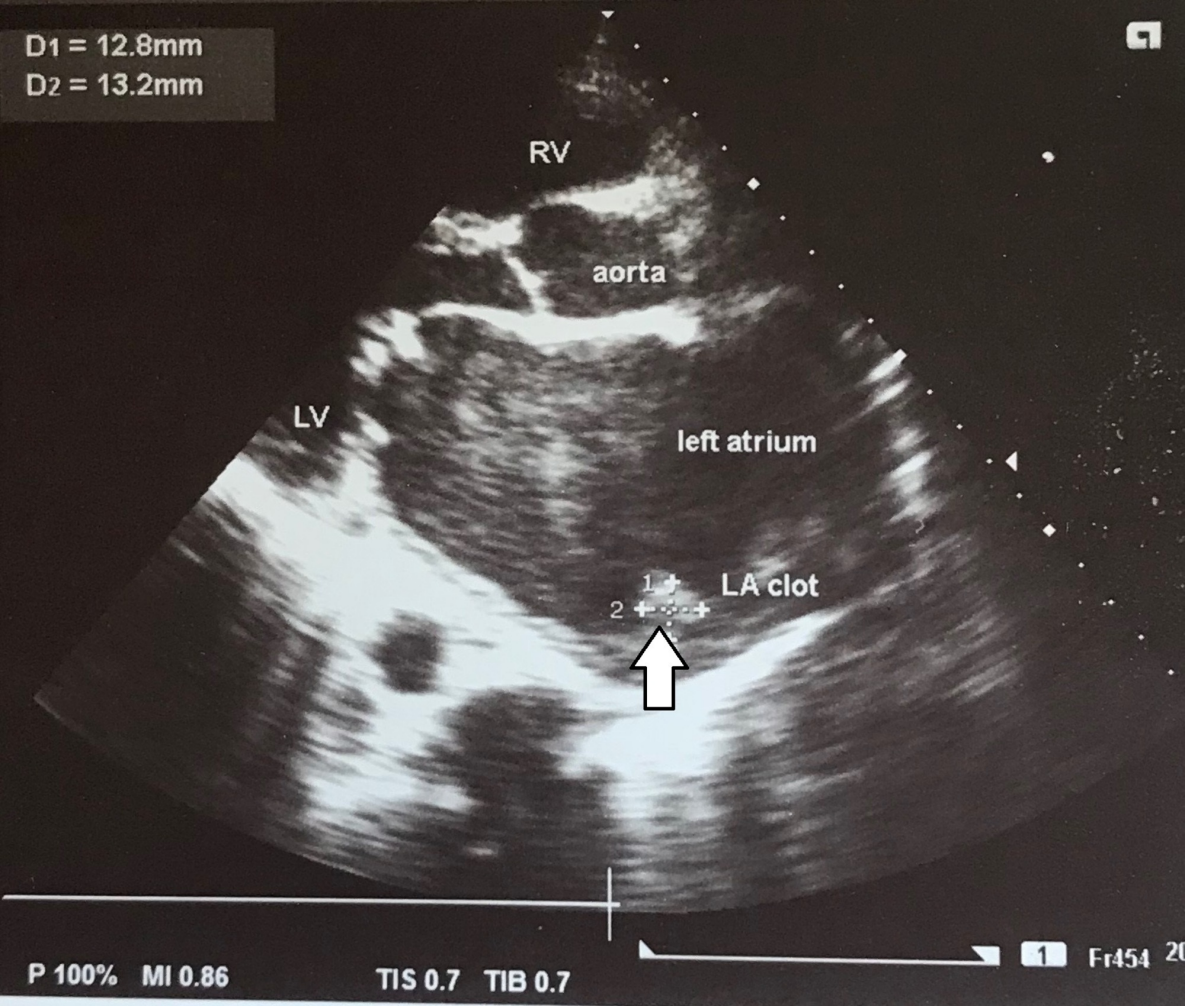
Transthoracic echocardiography on parasternal long axis view showing left atrial enlargement, left atrial clot measuring 12.8 mm x 13.2 mm (arrow), and thickened and calcified mitral valve LV: left ventricle; LA: left atrium; RV: right ventricle; D: diameter.

**Figure 5:**
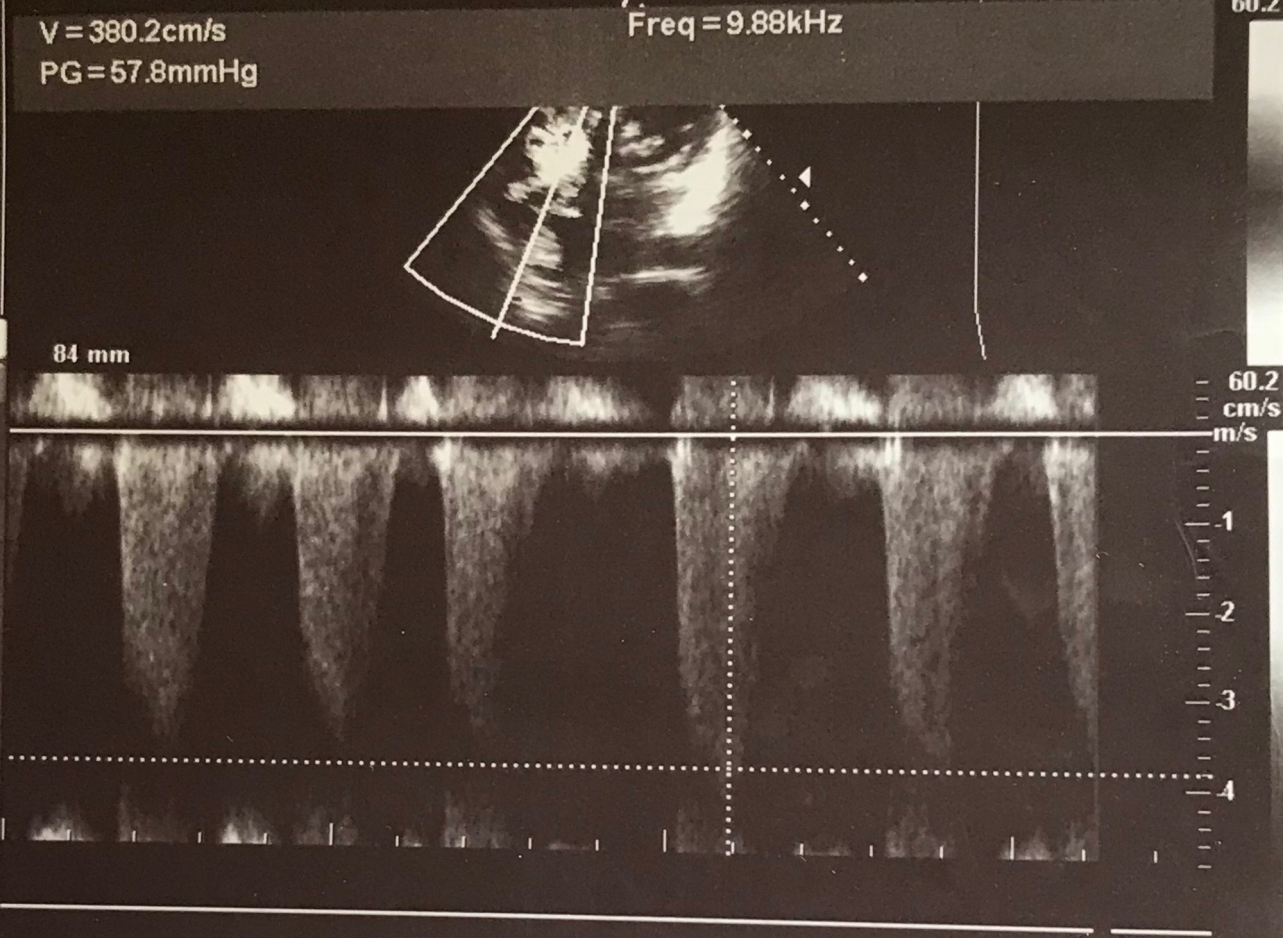
Transthoracic echocardiogram in four-chamber view with continuous wave (CW) mode showing severe tricuspid regurgitation with a pressure gradient of 57.8 mmHg V: velocity; PG: pressure gradient.

On investigation, electrocardiogram (ECG) showed ST segment elevation of more than two millimetre (mm) in chest leads V4, V5 and V6, suggesting ST elevation myocardial infarction (STEMI) and AF with normal ventricular rate (Figure 2). Transthoracic echocardiography showed thickened, calcified mitral valve leaflets with mitral valve area (MVA) of 1.01 cm^2^ on planometry, mitral valve pressure half-time (PHT) of 344 millisecond, and mean pressure gradient of 9 mmHg (features suggestive of rheumatic severe mitral stenosis), left atrial enlargement and a left atrial clot measuring 12.8 mm x 13.2 mm, severe tricuspid regurgitation (TR) (pressure gradient of 57.8 mm Hg), hypokinesia of antero-lateral wall of the left ventricle with left ventricle ejection fraction of 35% to 40% (Figures 3-5).

**Figure 6:**
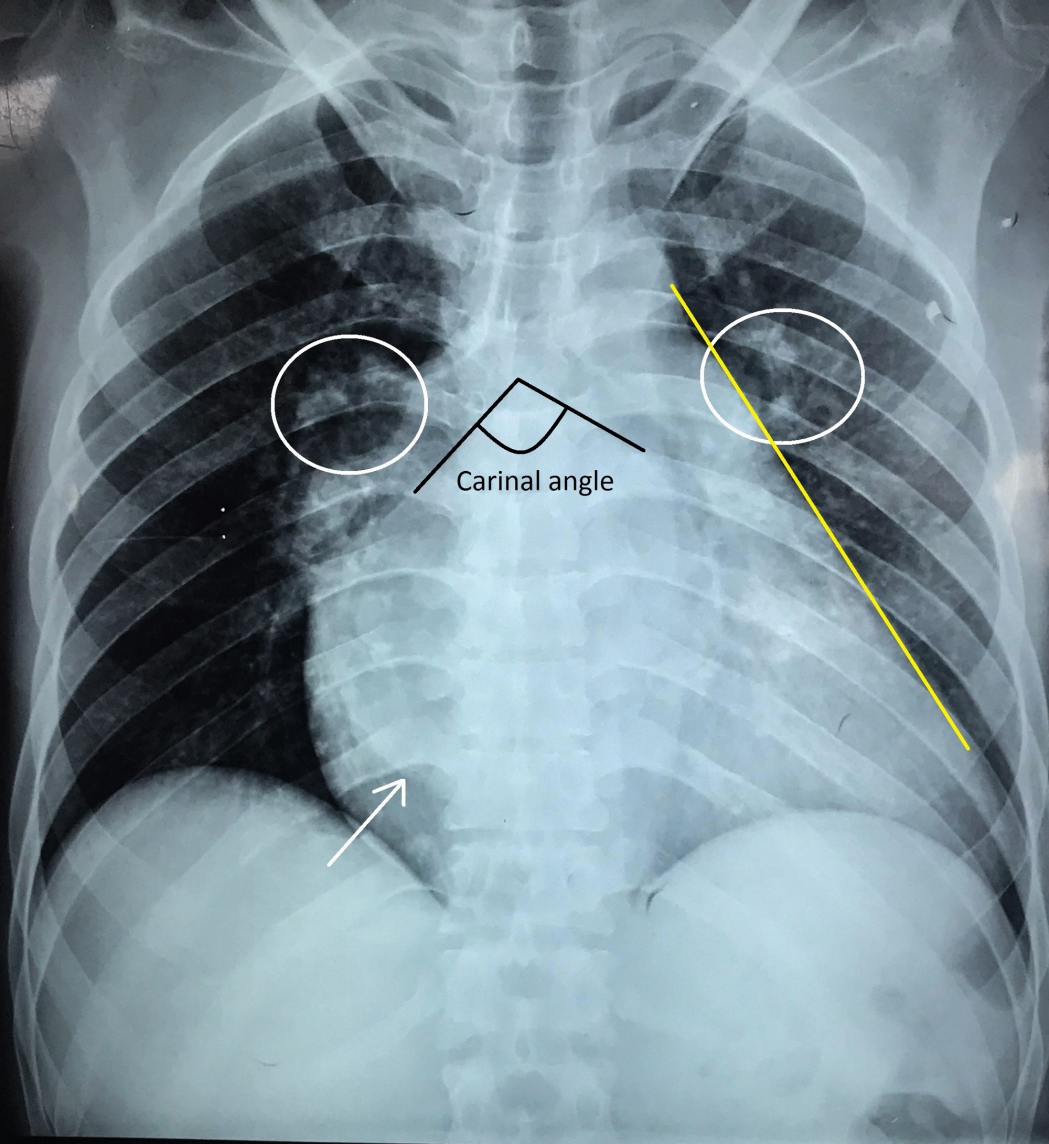
Chest radiograph (posteroanterior view) showing cardiomegaly with cardiothoracic ratio 70%, straightening of left heart border (yellow line), double right heart border (white arrow), cephalization (white circle), and widened carinal angle typical of left atrial enlargement The cardiac border was displaced laterally and downwards implying left ventricular enlargement.

Chest radiography revealed cardiomegaly with cardiothoracic ratio 70%, straightening of left heart border, double right heart border, cephalization (upturned moustache sign or Antler sign), and widened carinal angle (more than 90^o^) typical of left atrial enlargement. The cardiac border was displaced laterally and downwards implying left ventricular enlargement (Figure 6). 

**Figure 7:**
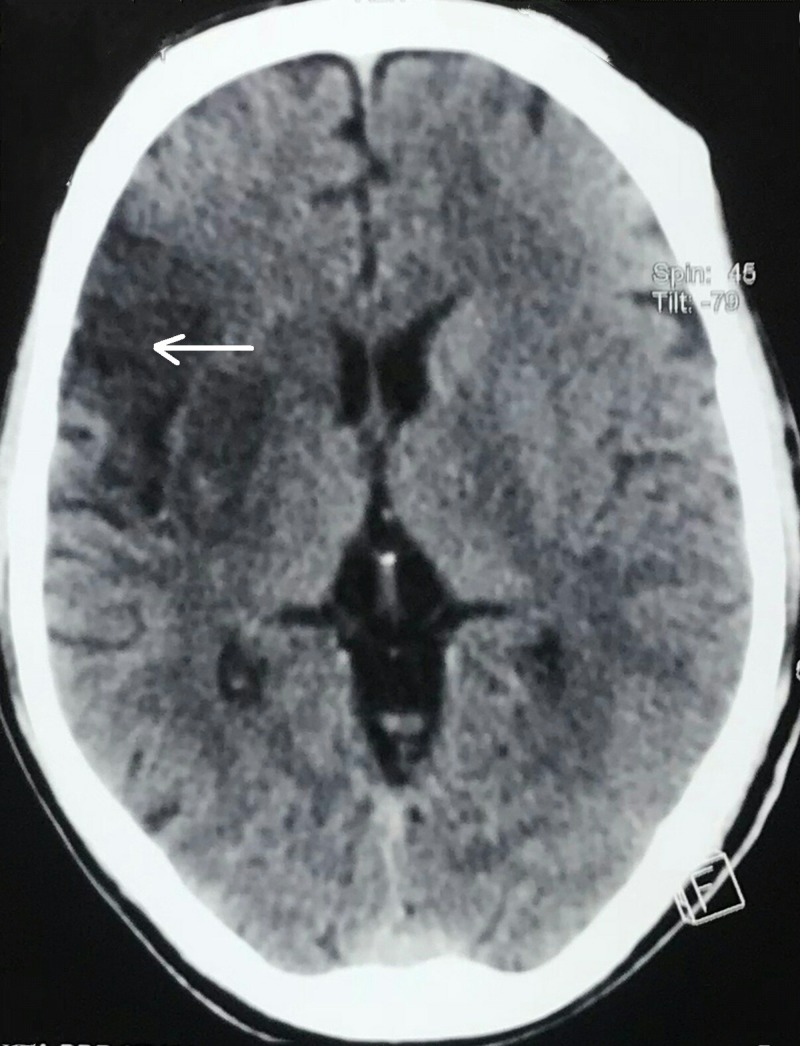
A computed tomography (CT) scan of the head showed right-sided ischemic stroke (white arrow) involving more than one-third of the middle cerebral artery territory

A computed tomography (CT) scan of the head showed right-sided ischemic stroke involving more than one-third of the middle cerebral artery territory (Figure 7).

Arterial doppler of the right leg showed nearly occluding echogenic content in right femoral artery with minimal triphasic flow and occluding echogenic content in right popliteal artery with no flow in color and spectral Doppler study. No flow was noted in the right tibioperoneal trunk, anterior tibial artery, posterior tibial artery, and dorsalis pedis artery. Venous Doppler of the right leg demonstrated deep vein thrombosis (DVT) involving right femoral and popliteal vein. Abdominal ultrasonography was normal.

At workup, the patient had total creatine phosphokinase (CPK) 10000 unit per liter (U/L) (normal= 10 to 120 U/L), creatine phosphokinase-MB (CPK-MB) 3000 U/L (normal= 0-25 U/L), troponin I 40.6 nanogram per mililiter (ng/ml) (positive for > 0.12 ng/ml), total white cell count 18,210 per microliter (uL) (normal= 4000-11000 per uL), hemoglobin 15.4 gram per deciliter (g/dl), creatinine 3.28 milligram per deciliter (mg/dl) (normal= 0.6 to 1.1 mg/dl), sodium 122 milliequivalent per liter (mEq/L) (normal= 135 to 145 mEq/L), potassium 6.2 mEq/L (normal= 3.5 to 4.5 mEq/L), total billirubin 1.4 mg/dl (normal is < 1 mg/dl), alanine trasaminase (ALT) 516 U/L (normal= 30 to 65 U/L), aspartate trasaminase (AST) 2030 U/L (normal= 0 to 45 U/L), alkaline phosphatase 117 U/L (normal= 40 to 140 U/L), total protein 6.8 g/dl (normal= 6.4 to 8.2 g/dl), albumin 3.2 g/dl (normal= 3.8 to 4.9 g/dl), and prothrombin time (PT) was 13.3 second with international normalized ratio (INR) 1.1 (control= 12 second). Serum ASO titer was not done as there was no active rheumatic activity.

A provisional diagnosis of RHD with severe MS and TR with AF leading to multiple systemic embolizations was formulated. Anticoagulation was started with low molecular weight heparin (enoxaparin 60 mg subcutaneous twice daily). The patient received aspirin 75 mg and digoxin 0.125 mg once daily. Hyperkalaemia was managed medically with intravenous calcium gluconate one gram over 10 minutes, 50 ml of 50% dextrose with regular insulin of 10 U and salbutamol nebulization. Subsequently, the patient underwent above knee amputation of the right lower limb. His liver and kidney function tests were monitored until they were normalized. He was discharged on warfarin 6 mg, aspirin 75 mg, spirinolactone 25 mg, enalapril 2.5 mg, digoxin 0.125 mg once daily and frusemide 20 mg twice daily. The patient was adviced for regular follow-up for monitoring his clinical status and adherence to medications. He paid two visits (one week apart) to the hospital after discharge and his clinical conditions were improving.

## Discussion

Of all native valvular diseases, rheumatic mitral valve disease carries the highest risk of systemic embolization. The incidence of AF in patients with RHD is 43.61% [2]. The presence of AF increases the risk of systemic embolization and mitral stenosis carries a higher risk of embolization compared with mitral regurgitation [2]. After the first episode of embolization, recurrent embolization occurs in 30%-65% of patients; with more than half of recurrences occurring during the first one year [3]. Similarly, in our case, the cardioembolic phenomenon associated with RHD was the prime cause of the patient's illness.

The non-valvular atrial fibrillation (NVAF) increases the risk for ischemic stroke by five-fold, the risk is even greater among patients with mitral stenosis, increasing up to 17-fold [4]. Our patient had severe MS with AF and was at increased risk of cardioembolic stroke. Stroke in patients with AF is generally more severe and the outcome is markedly poorer than in patients with sinus rhythm. Studies in patients with both rheumatic mitral stenosis and atrial fibrillation have shown that warfarin is effective in preventing cerebral embolism [5]. Mitral valve repair and mitral valve replacement are also considered in the prevention of cerebral embolism for patients with hemodynamically significant rheumatic mitral stenosis or insufficiency [3].

When the afflicted patients are young, the tragic consequences for family, friends, and occupation are particularly catastrophic and unexpected as with our case. In a developing country like Nepal, where there is no facility for regular monitoring of PT/INR, anticoagulation with warfarin may not be feasible. However, the evidence regarding the use of newer oral anticoagulants like rivaroxaban and dabigatran in rheumatic valvular AF is yet to be proven. In this case, we discharged the patient with warfarin as an anticoagulant with provision for regular monitoring of PT/INR risking the adherence of the patient with it.

RHD with mitral stenosis presenting for the first time as acute STEMI is rare. In a prospective study (n=376) conducted by Jose VJ et al. [6], the overall prevalence of coronary artery disease in a group of patients with rheumatic heart disease undergoing valve surgery was 12.2%. In our case, the patient was diagnosed with STEMI based on ECG changes, cardiac biomarkers, and echocardiography, however, he didn’t give a history of chest pain. Primary percutaneous coronary intervention (PPCI) was not performed in this patient as there was no evidence of ongoing myocardial ischemia and no features suggestive of heart failure or cardiogenic shock.

Acute peripheral arterial occlusion is characterized by "the six Ps": pain of sudden onset, pallor, pulselessness, paresthesias, paresis, and prostration with the symptoms of shock. Femoral bifurcation, the superficial femoral artery, and the popliteal artery are the most common sites for lodgment of embolus [7] and diseased heart is the main source of arterial emboli [8]. Our case is in accordance with the above-mentioned studies. Surprisingly, our patient also suffered from DVT in the right limb, which is an unusual finding. This might have arisen due to a restriction of movement in the right limb caused by ischemic pain.

Apart from cerebral, coronary and peripheral arterial embolisms, such patients are also vulnerable for renal and mesenteric embolisms. Renal infarction mostly presents as abdominal pain and hematuria with an evident rise in serum lactate dehydrogenase [9].

Renal impairment was present in our case and the possible causes of renal impairment could be renal ischemia or rhabdomyolysis of the right ischemic limb. Rhabdomyolysis is more likely to be the cause of renal impairment in our case as there was evidence of hyperkalaemia, raised CPK and liver enzymes. In 50% of cases of rhabdomyolysis, urine could be normal in color, as with our case. Though the ultrasonography of the abdomen was normal, CT angiography of the abdomen to rule out renal ischemia was not performed in our case. After amputation of the ischemic limb, renal impairment and liver enzymes normalized.

Rheumatic heart disease is effectively controlled by long-term antibiotic prophylaxis. Duration of therapy depends on the severity of cardiac involvement. Lifelong anticoagulation is the mainstay of prophylaxis for a cardioembolic event in patients of mitral stenosis with atrial fibrillation. This is, to the best of our knowledge, the first case report of it's kind in medical literature.

## Conclusions

Multiple systemic embolizations must always be considered in patients with valvular heart disease, especially in MS associated with AF. Clinical suspicion and follow up of such patients confirming adherence to prophylaxis can prevent adverse outcomes.
